# New Adenovirus Groups in Western Palaearctic Bats

**DOI:** 10.3390/v10080443

**Published:** 2018-08-20

**Authors:** Maria Iglesias-Caballero, Javier Juste, Sonia Vázquez-Morón, Ana Falcon, Carolina Aznar-Lopez, Carlos Ibáñez, Francisco Pozo, Guillermo Ruiz, Jose M. Berciano, Inazio Garin, Joxerra Aihartza, Juan E. Echevarría, Inmaculada Casas

**Affiliations:** 1 Centro Nacional de Microbiología, Instituto de Salud Carlos III, Carretera de Majadahonda-Pozuelo km 2. Majadahonda 28220, Madrid, Spain; miglesias@isciii.es (M.I.-C.); svazquez@isciii.es (S.V.-M.); afalcon@cnb.csic.es (A.F.); carolinaaznarlopez@hotmail.com (C.A.-L.); pacopozo@isciii.es (F.P.); guillermo.ruiz@salud.madrid.org (G.R.); jmberciano@isciii.es (J.M.B.); jeecheva@isciii.es (J.E.E.); 2 Estación Biológica de Doñana, CSIC, Avda Américo Vespucio 16, 41092 Seville, Spain; juste@ebd.csic.es (J.J.); ibanez@ebd.csic.es (C.I.); 3 Centro de Investigación Biomédica Epidemiología y Salud Pública, CIBERESP, 28029 Madrid, Spain; 4 Consorcio Centro de Investigación Biomédica en Red (CIBER), 28029 Madrid, Spain; 5 Department of Zoology and Animal Cell Biology, University of the Basque Country (UPV/EHU), Leioa 48940, Basque Country, Spain; inazio.garin@ehu.eus (I.G.); joxerra.aihartza@ehu.eus (J.A.)

**Keywords:** Adenovirus, Western Palaearctic Bats, Phylogenetic analysis, Spain

## Abstract

In the context of long-term screening for viruses on Western Palaearctic bats, we tested for the presence of adenovirus 1392 oropharyngeal swabs and 325 stool samples taken from 27 bat species. Adenoviruses were detected in 12 species of the Vespertilionidae and the Rhinolophidae families. Fifty positive respiratory and 26 positive stool samples were studied. Phylogenetic analyses of partial hexon protein and partial DNA-dependent DNA polymerase genes indicate that all these bat adenoviruses belong to the genus *Mastadenovirus* but without constituting a monophyletic cluster. According to genetic identities, the new groups are distinct to the previously described *Bat mastadenovirus A* and *B* species and contribute with potentially new members. Our data support that diversity of bat mastadenovirus is host-dependent and increase the knowledge of potentially pathogenic virus from bats. Due to the active role of bats as viral reservoirs, the characterization of these viruses is relevant for Public Health.

## 1. Introduction

Bats are the second largest order of mammals, comprising more than 1200 different species [1]. Their high vagility and the organization typically in social groups predispose them to infection and viral dissemination [2]. Extensive surveys have shown their susceptibility to host a wide range of viruses and the possibility to be a source of emerging infectious in humans [3]. The Order Chiroptera plays a role as a reservoir for many significant virus families such as *Rhabdoviridae*, *Coronaviridae*, *Herpesviridae*, *Filoviridae*, *Reoviridae*, *Paramyxoviridae* and *Astroviridae*, among others. Several studies have shown bats to be a reservoir of Adenoviruses [4,5,6,7].

Adenoviruses (AdVs) are subdivided in five genera, *Mastadenovirus* (mammals), *Aviadenovirus* (birds), *Atadenovirus* (mammals, birds and reptiles), *Siadenovirus* (birds and amphibians) and *Ichtadenovirus* (fish) [8]. In 2008, the first AdV from a bat, BtAdV1-FBV1, was isolated during attempts to establish a specific cell line from a Ryukyu flying fox (*Pteropus dasymallus yayeyamae*), in Japan [9]. Following a screening of 55 German free-ranging bats, family Vespertilionidae, a second, BtAdV-2 strain PPV1, was identified in 3 common pipistrelles (*Pipistrellus pipistrellus*) [10], being the first AdV isolated from a microchiropteran bat and the second fully sequenced genome [11]. The first fully sequenced AdV genome from a bat was the BtAdV-3 strain TJM from a Rickett’s big-footed bat (*Myotis ricketti*) [12]. According to ICTV, BtAdV-3 strain TJM and BtAdV-2 strain PPV1 were renamed as *Bat mastadenovirus A* and *B*. Several other studies have shown a large genetic viral diversity in bats from Brazil [13], Japan [9], Germany [4,10,11,14], China [12,15], Hungary [5,14], Ghana [16], Zambia [17], Kenya [7], South Africa [18] and USA [19].

As a consequence of these studies, the following viruses are candidates for the ICTV to be novel bat species in the future: *Bat mastadenovirus C* (*Rhinolophus sinicus* WIV9, KT698853 ) [6], *Bat mastadenovirus D* (*Miniopterus schreibersi* WIV12, KT698856) [15], *Bat mastadenovirus E* (*M. schreibersi* WIV13, KT698852) [15], *Bat mastadenovirus F* (*Rousettus leschenaultii* WIV17, KX961095) [15] and *Bat mastadenovirus G* (*Corynorhinus rafinesquii* 250-A, KX871230) [19].

In Spain, rabies surveillance has become an important issue due to its geographic position between Africa and Europe [20], particularly bats with expected genetic flow between the South of Spain and the North of Morocco such as *Eptesicus isabellinus* [21]. Several studies have confirmed both Iberian species of Eptesicus as rabies vectors [22,23] including the detection of the new Lleida bat lyssavirus [24]. Other studies have described new viruses, such as a novel Lloviu filovirus detected in dead *Miniopterus schreibersii* in the North of Spain [25], 14 coronavirus distributed in new groups including two betacoronavirus related with the MERS-CoV group [26], 42 potentially novel betaherpesvirus and 10 potentially novel rhabdovirus from the families Vespertilionidae, Miniopteridae, Rhinolophidae, Molosidae and Pteropodidae in the South and North of Spain [27,28]. These studies have increased the knowledge of new viruses and their potential as human pathogens. Human AdVs cause a wide range of clinical syndromes and are being increasingly recognized in cases of severe or fatal pneumonia, haemorrhagic cystitis, hepatitis, or disseminated disease in paediatric bone marrow transplant recipients. Due to the active role of bats as viral reservoirs, this knowledge is an important part of the Public Health surveillance. Our study aimed to investigate the AdVs circulating in bats to describe their phylogenetic relationship by analysing two distinct informative partial genes.

## 2. Materials and Methods

### 2.1. Origin of Samples and Preparation

**Figure 1:**
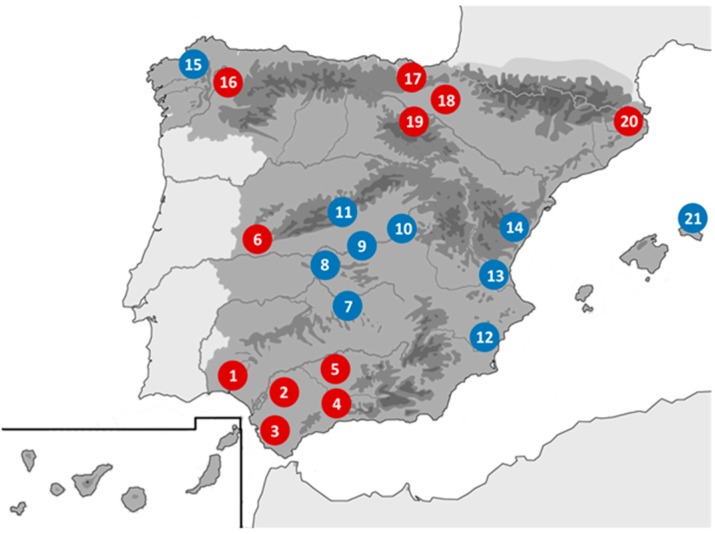
Geographical distribution of Bat capture locations in Spain. South of Spain: 1. Huelva, 2. Seville, 3. Cádiz, 4. Málaga, 5. Córdoba. Centre of Spain: 6. Cáceres, 7. Ciudad Real, 8. Toledo, 9. Madrid, 10. Guadalajara, 11. Segovia, 12. Alicante, 13. Valencia, 14. Castellón. North of Spain: 15. A Coruña, 16. Lugo, 17. Biscay, 18. Navarra, 19. La Rioja, 20. Gerona. Balearic Islands: 21. Menorca. Red circles are locations with AdV positive samples.

During 2004 to 2016, in the context of rabies surveillance, a screening for other different viruses was performed according to the General Research Program protocol of the Spanish Government (specific projects SAF2006-12784-C02/01-02, SAF2009-09172 and SAF2013-47194-P, approved on 10 January 2006, 20 November 2009 and 3 December 2013, respectively). Bats were captured and sampled in several campaigns across the Iberian Peninsula (Figure 1). Sampling methods followed the regulations and ethical procedures of the Spanish Bat Society (SECEMU). After being captured, each animal was identified, sexed, measured and weighed. For identification of cryptic species complexes, a wing-punch sample was collected for analysis of a cytochrome-b gene fragment [21]. For virological studies, oropharyngeal swabs (OPS) and stool samples (SS) were collected and homogenized in 1 mL of lysis buffer. After being studied and sampled, bats were released at the same location. Samples were sent to the Rabies National Reference Laboratory, aliquoted and stored at −80 °C until tested. Total nucleic acids were extracted from aliquots of 200 µL-buffered suspension and pellets were diluted in 50 µL of water [29].

### 2.2. Adenovirus Detection by Generic PCR Methods

Two independent generic PCR assays were used for the detection of members of the family *Adenoviridae*. A panAdVHex nested PCR that amplified one of the seven hypervariable regions of the hexon gene and had previously been using for human AdVs genotyping was used for screening of samples [30,31]. Five μL of nucleic acids extracted were added to 45 μL of reaction mixture containing 60 mM Tris-HCl (pH 8.5), 15 mM (NH_4_)_2_SO_4_, 0.4 mM each of dNTPs (GE Healthcare, Buckinghamshire, UK), 60 pmol of each primer and 2.5 U AmpliTaq DNA Polymerase (Applied Biosystems, Branchburg, NJ, USA). Cycling conditions were: 95 °C-4 min and 40 cycles, 95 °C-30 s, 50 °C-2 min, 72 °C-30 s. For nested reactions, same reagents and cycling conditions were used. Amplified products (~768 bp) were visualized following 2% agarose gel electrophoresis. To increase the phylogenetic accuracy, a panAdVPol hemi-nested PCR assay targeting a taxonomical informative fragment of the DNA-dependent DNA polymerase gene (DNApol) was designed and used. Five μL of extract was added to 20 μL of reaction mixture (LightCycler 480, Roche Diagnostics, Mannheim, Germany) and 10 pmol of the primers pol-F (5’GTIGCRAAIGAICCRTAGAGGGC 3’) and pol-R (5’GTTTAYGAYATITGYGGMATGTAYGC 3’). The amplification conditions were: 95 °C-5 min, followed by 45 cycles, 95 °C-15 s, 57 °C-2 min, 68 °C-30 s. For heminested reactions, 2 µL of the previously amplified DNA and 10 pmol of the primers pol-F2 (5’AAIGAICCRTAGAGGGCRTTKGA 3’) and pol-R were added to a reaction mixture containing 60 mM Tris-HCl (pH 8.5), 15 mM (NH_4_)_2_SO_4_, 0.2 mM each of dNTPs and 1.25 U AmpliTaq DNA Polymerase. The amplification conditions were: 95 °C-5 min, followed by a two-step-cycle of 95 °C-15 s and 62 °C-2 min 45 times. Amplified products (~450 bp) were visualized following electrophoresis on a 2% agarose gel.

### 2.3. Sequence and Phylogenetic Analysis

Purified amplified products of the expected size were double-strand sequenced by Sanger chain-termination method using the BigDye Terminator v3.1 Cycle Sequencing Kit in an ABI PRISM 3700 DNA Analyzer (Applied Biosystems). The nucleotide sequences were compared with those published in GenBank database using the BLASTn algorithm (http://blast.ncbi.nlm.nih.gov/) to assess and identify similar AdV sequences. Two nucleotide multiple-sequence alignments from the hexon and DNApol genes, comprising a selection of available mastadenovirus sequences from the GenBank database, were constructed using CLUSTAL X (v.2.0; http://www.clustal.org/). Phylogenetic analysis was performed with MEGA 5.2 software (http://www.megasoftware.net) and were based on a Neighbour-Joining criterion using a Tamura 3 and Kimura 2-parameter models for the hexon and DNApol genes respectively, selected by Modeltest software [32]. Pairwise distance comparison between the predicted DNApol amino acid sequences of Iberian bat AdVs and *Bat mastadenovirus A* and *B* was calculated using MEGA 5.2 software. Names for the putative new bat AdVs were assigned using the bat host species abbreviation and the identification ring number.

## 3. Results

Bat species studied, year of capture, type of sample and the corresponding GenBank accession numbers for the Iberian bat AdV sequences are listed in Table 1.

We screened a total of 1717 samples, 1392 OPS and 325 SS, representing 27 out of the 32 European bat species (http://secemu.org), belonging to the families Vespertilionidae (22 sspp), Miniopteridae (1 sp) and Rhinolophidae (4 sspp). AdV DNA was detected in 50 OPS (3.6%) and in 26 SS (8.3%). Seventy individual bats had detectable levels of AdV DNA with three of these being positive in both OPS and SS. Successful amplification of the partial AdV hexon gene was obtained in 69 samples, (49 OPS and 20 SS) and for the partial DNApol gene in 35 samples (14 OPS and 21 SS). All amplified products were confirmed by sequencing and individual sequences were deposited in the GenBank database (Table 1). In 29 bats both partial genes were studied. In 41 bats only the hexon sequence were obtained. Finally, in 6 bats only the DNApol was studied.

The Andalusia region in the south of Spain, had the greater distribution of AdV in bats including several genera of the families Vespertilionidae, (*Pipistrellus*, *Myotis* and *Nyctalus*) and the Rhinolophidae (*Rhinolophus*). The majority of positive bats belonging to the *Pipistrellus* genus were sampled in Andalusia. All 59 bat AdVs found in the *Rhinolophus* genus also came from Andalusian bats while no positives were detected in 78 bats sampled in the Basque Country (North). The three *Nyctalus* species (*N. noctula*, *N. lasiopterus* and *N. leisleri*, 23 bats) and two of the three *Pipistrellus* (*P. kuhlii* and *P. pygmaeus*, 28 bats) contributed the most to the list of positives detected in OPS and SS. Two out of four species of the Rhinolophus genus (*R. euryale* and *R. ferrumequinum*, 14 bats) had detectable levels of AdV DNA present in the OPS samples only.

### 3.1. Phylogenetic Analysis of Bat AdV Sequences

**Figure 2:**
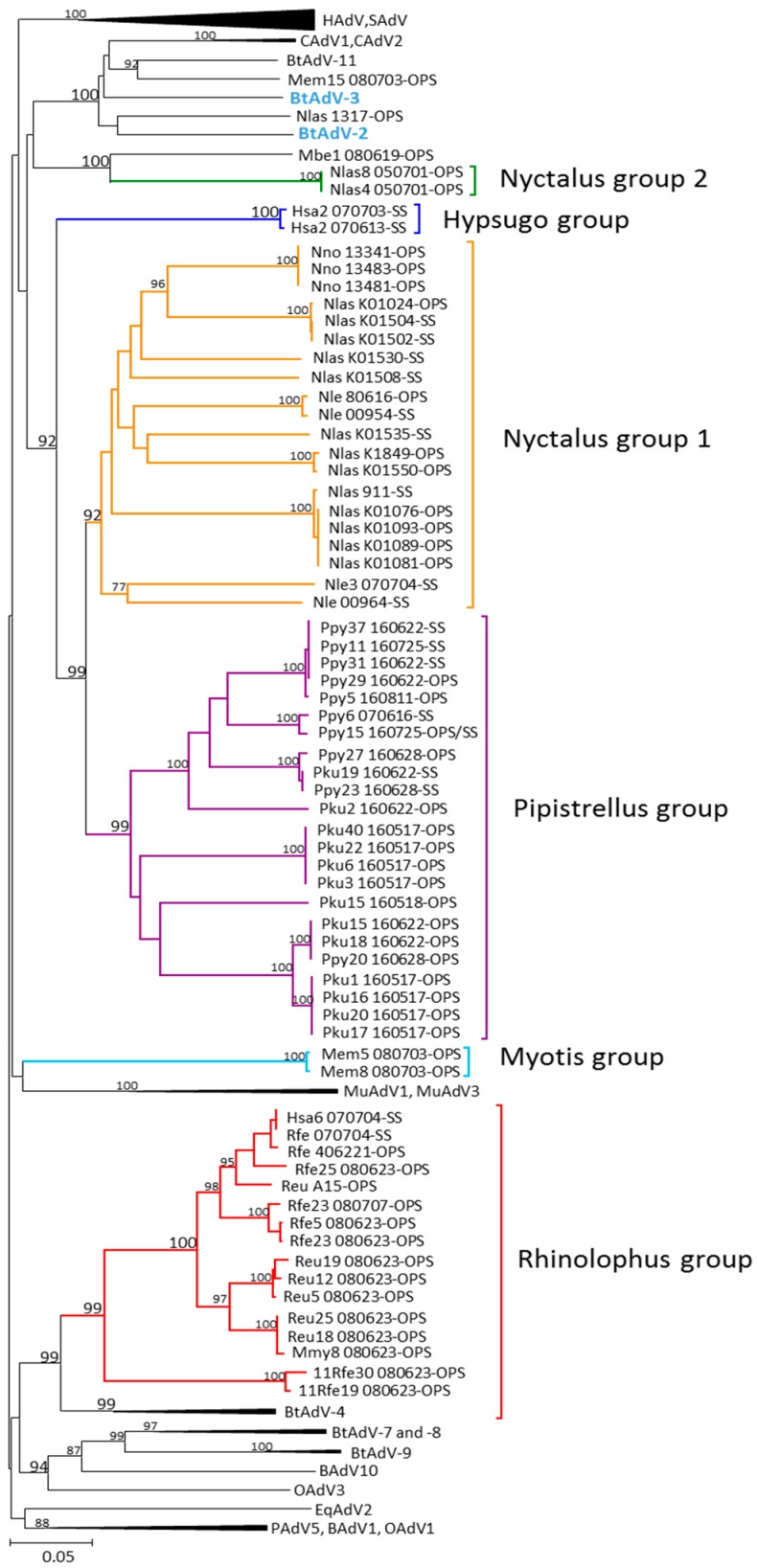
Phylogenetic tree based on the analysis of the hexon partial gene. Trees were estimated with MEGA 5.2 software by using the neighbour-joining method on Tamura 3 parameters model. A bootstrap test was replicated for 5000 times. Numbers represent percentage bootstrap support. GenBank accession numbers for the sequences included in the tree are as follows: BtAdV-3 (strain TJM, GU226970, *Bat mastadenovirus A*), BtAdV-2 (strain PPV1, JN252129, *Bat mastadenovirus B*), human AdVs: type 1 (AF534906), type 2 (J01917), type 3 (DQ086466), type 4 (AY594254), D8 strain Ger/Berlin/04_2003 (KT862545), type 9 (AJ854486), type 12 (X73487), type 14 (FJ841902), type 16 (X74662), type 21 (KF528688), type 24 (JN226751), type 27 (JN226753), type 42 (JN226761), type 45 (JN226764), simian AdVs: type 1 (AY771780), type 4 (KP853121), ovine AdVs: type 1 (DQ630754), type 3 strain (DQ630756), porcine AdV 5 (AF289262), murine AdVs: type 1 (M81889), type 3 (EU835513), bovine AdVs: type 1 (DQ630761), type 10 (AF282774), canine AdVs: type 1 (KX545420), type 2 (U77082), equine AdV type 2 (L80007), bat mastadenovirus: BtAdV-7 (strain WIV12, KT698856, *Bat mastadenovirus D*), BtAdV-8 (strainWIV13, KT698852, *Bat mastadenovirus E*), BtAdV-9 (strain WIV17, KX961095, *Bat mastadenovirus F*), *Rousettus leschenaultii* WIV18 (NC_035072), BtAdV-4 (strain WIV9, KT698853, *Bat mastadenovirus C*), *Rhinolophus sinicus* WIV10 (NC_029899), *R. sinicus* WIV11 (NC_029902), BtAdV-11 (strain 250-A, KX871230, *Bat mastadenovirus G*).

**Figure 3:**
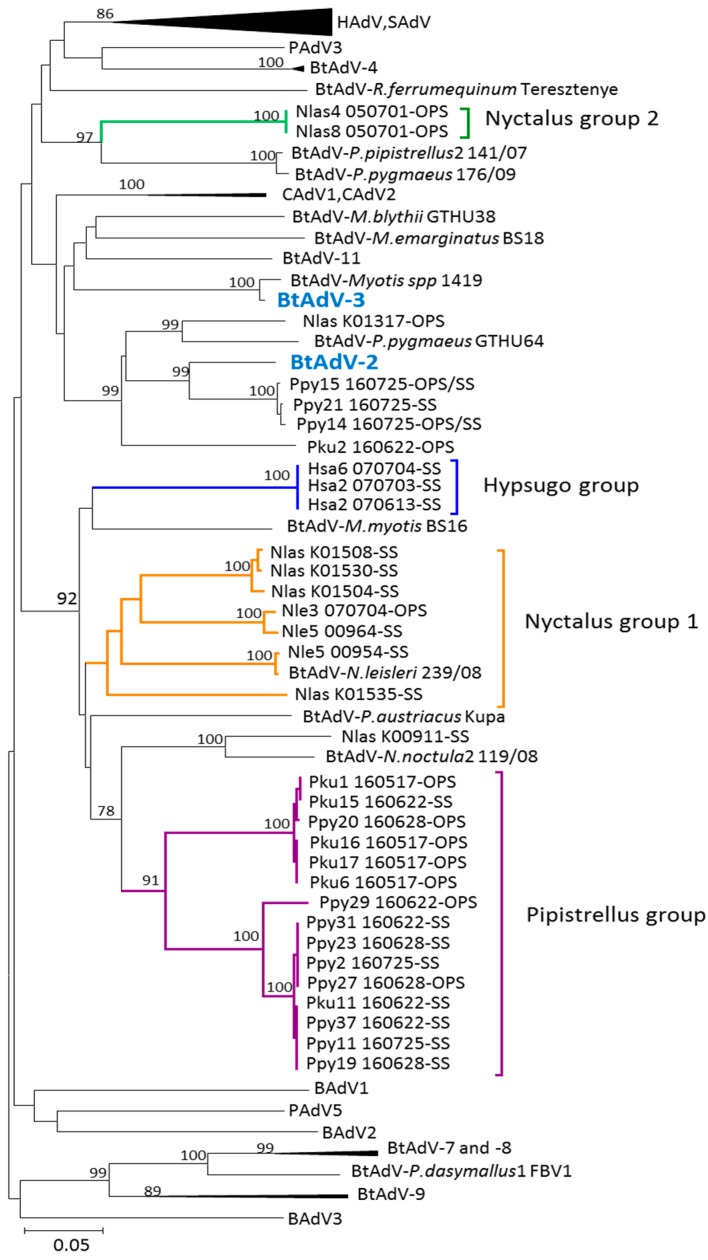
Phylogenetic tree based on the analysis of the DNA-dependent DNA polymerase partial gene. Trees were estimated with MEGA 5.2 software by using the neighbour-joining method on Kimura 2 parameters model. A bootstrap test was replicated for 5000 times. Numbers represent percentage bootstrap support. GenBank accession numbers for the sequences included in the tree are as follows: BtAdV-3 (strain TJM, GU226970, *Bat mastadenovirus A*), BtAdV-2 (strain PPV1, JN252129, *Bat mastadenovirus B*), human AdVs: type 1 (AF534906), type 2 (J01917), type 3 (DQ086466), type 4 (AY594254), type 5 (AY339865), type 7 (AY594256), type 6 (HQ413315), type 9 (AJ854486), type 12 (X73487), type 17 (AF108105), type 19 (JQ326209), type 26 (EF153474), type 48 (EF153473), type 53 (AB605245), simian AdVs: type 1 (AY771780), type 4 (KP853121), bovine AdVs: type 2 (AF252854), type 3 (AF061654), type 1 (AC_000191), porcine AdVs: type 3 (AB026117), type 5 (AF289262), canine AdVs: type 1 (KX545420), type 2 (U77082), bat mastadenovirus: BtAdV-7 (strain WIV12, KT698856, *Bat mastadenovirus D*), BtAdV-8 (strainWIV13, KT698852, *Bat mastadenovirus E*), BtAdV-9 (strain WIV17, KX961095, *Bat mastadenovirus F*), *Rousettus leschenaultii* WIV18 (NC_035072), BtAdV-4 (strain WIV9, KT698853, *Bat mastadenovirus C*), *Rhinolophus sinicus* WIV10 (NC_029899), *R. sinicus* WIV11 (NC_029902), BtAdV-11 (strain 250-A, KX871230, *Bat mastadenovirus G*), *Plecotus austriacus* Kupa (JN167523), *Rhinolophus ferrumequinum* Teresztenye (JN167522), *Myotis spp 1419* (GU226962), *R. leschenaultii* 1050597 (HQ529709), *Nyctalus noctula* 2 119/08 (KM043096), *Myotis emarginatus* BS18 (KM043084), *Myotis myotis* BS16 (KM043106), *Myotis blythii* GTHU38 (KM043086), *Nyctalus leisleri* 239/08 (KM043102), *Pipistrellus pygmaeus* GTHU64 (KM043090), *P. pygmaeus* 176/09 (KM043091).

Our sequences from the partial AdV hexon and DNApol genes, Figure 2 and Figure 3 respectively, were included within the genus *Mastadenovirus*. High bootstrap values supported clusters which differentiate the bat mastadenovirus from the families Rhinolophidae and Vespertilionidae. Similar clustering in the phylogenetic trees using the partial hexon and DNApol genes were observed when compared to the complete genomes sequences with high bootstrap values obtained.

### 3.2. Partial AdV Hexon Gene Sequence Analysis

Two of the 73 bat AdVs detected in OPS from a *Nyctalus lasiopterus* (HM856343) and a *Myotis emarginatus* (MF540610) slightly related with bat adenovirus 11 (species *Bat mastadenovirus G*) (KX871230) from a *Corynorhinus rafinesquii* captured in USA [19], clustered with the reference strains bat adenovirus 2 (JN252129) and 3 (GU226970), detected in a *Pipistrellus pipistrellus* from Germany [10] and in a *Myotis ricketti* from China [12], respectively.

The rest of the 73 bat AdVs clustered in six groups with significant bootstrap values, supporting potential novel groups within the genus *Mastadenovirus* based on the bat species. These new groups are host differentiated: *Pipistrellus* group (Table S1), *Nyctalus* group 1, *Nyctalus* group 2 (Table S2), Hypsugo group and Myotis group (Table S3) of the Vespertilionidae family and a Rhinolophus group (Table S4) of the Rhinolophidae family (Figure 2 and Figure 3).

*Nyctalus* group 1 represented a cluster of 13 AdVs from *N. lasiopterus*, four from *N. leisleri* and three from *N. noctula*, highly associated with the *Pipistrellus* group. *Nyctalus* group two AdVs clustered apart including two AdVs from two distinct *N. lasiopterus* and one from a *Myotis bechsteinii* (MF540611). In a well-defined *Pipistrellus* group (bootstrap 99) 13 AdVs cluster from *P. kuhlii* and 9 from *P. pygmaeus*. Similarly, the well-defined Rhinolophus group included eight from *R. ferrumequinum* and six from *R. euryale* and two others from a *Myotis emarginatus* (MF540609) and one from *Hypsugo savii* (HM856338). This cluster was highly supported and included three bat AdVs detected in *R. sinicus* captured in China [6]. Furthermore, two distinct AdV detected from two *Hypsugo savii* bats were grouped in one independent cluster defined as Hypsugo group was highly related with the *Nyctalus*-group 1 and the *Pipistrellus* group. Additionally, two AdVs detected in two *Myotis emarginatus* bats constituted a new Myotis group (Figure 2).

### 3.3. Partial DNA-Dependent DNA Polymerase Gene Sequences

The groups defined in this gene were clearly associated by host with lower support in some nodes and less resolution compared with the hexon partial gene analysed (Figure 3).

Five AdVs detected in *Pipistrellus pygmaeus* (MF404968, MF404979, MF404969, MF404988 and MF404978) clustered together with the reference *bat adenovirus 2* (JN252129) in a group which included three AdVs detected in a *Pipistrellus kuhlii* (MF404975), in a *Nyctalus lasiopterus* (JX065123) and in a *P. pygmaeus* (KM043090) captured in Hungary [14]. This group, which included 6 AdVs found in the genus *Pipistrellus*, grouped separately from the rest of our *Pipistrellus* bat AdVs.

Sequences from the genus *Nyctalus* grouped similarly with those defined in the hexon gene with the exception of a *N. lasiopterus* (JX065117) from the group 1 which was associated with an AdV detected in a *N. noctula* (KM043110) from Hungary. The *Nyctalus* group two was clustered with two different detected in a *P. Pipistrellus* (KM043096) and in a *P. pygmaeus* (KM43091) from Hungary. The *Pipistrellus* group which contained five AdVs detected in *P. kuhlii* and 12 in *P. pygmaeus* clustered together to define a similar group to that observed with the partial hexon gene analysis. In the Hypsugo group a *H. savii* (MG208122) clustered in the Hypsugo group unlike in the hexon gene analysis where this AdV clustered in the Rhinolophus group. No positive results were obtained in this gene with the rhinolophid bats.

Pairwise distance matrix values obtained from the partial amino acid sequence of DNApol, supported the new groups (Table 2). According to the pairwise distance data none of the Iberian bat AdVs were related with the species *Bat mastadenovirus A.* Likewise, three AdVs detected in *Pipistrellus pygmaeus* (Ppy14_160725, Ppy15_160725 and Ppy21_160725) had an amino acid pairwise distance of 12% being these viruses similar to the species *Bat mastadenovirus B*.

## 4. Discussion

In this work, we describe the detection and the phylogenetic relationships among potentially new bat mastadenoviruses and known AdVs from bats using two different partial genes. Our study shows, for the first time, their diversity in bats captured in the South of Europe and particularly in Spain a region of crucial importance for its strategic geographical placement, as a corridor between Africa and Europe.

Previous studies have shown a high diversity of AdVs found in bat species analysed across Europe, Asia and Africa [9,10,13,15,17,19]. In this study of AdV in bats, 27 out of the 32 Iberian bat species were examined obtaining positive results in 12 species from 6 bat genera. In Centre of Europe, Hungary and Germany, have also found positive results for AdVs in 9 of these 12 species [14]. With the aim of having a broad representation of the AdV diversity in the Iberian bats, a total of 1717 biological samples were analysed representing the largest AdV screening of bats for adenovirus. These bats were captured within Spain in a variety of habitats, from the Pyrenees and Cantabrian mountain ranges in the North to the Mediterranean South, considered as natural border with Africa and including several bat species with possible gene flow across the Gibraltar Strait [33].

The percentage of AdV positive bats was 3.6% in OPS and 8.3% in SS over the 18.6% in German samples and the 9.9% in Hungary [14]. These marked differences could be explained by the health of the bats and/or the use of different type of biological samples, from the homogenised internal organ tissues taken in dead or injured bats in the German study to healthy bats and guano samples in roosting places in the Hungarian. Positive bat AdV percentage similarity between our study and the Hungarian could be explained by the type of samples studied (OPS and SS). It is noteworthy the absence of AdVs in some bats such as the bent-winged *Miniopterus schreibersii*, despite the large number of individuals of this species screened. Similar negative results were found in Germany and Hungary [14]. Most of the AdV positive bats were found within the diverse bat family Vespertilionidae and particularly within the tribe *Pipistrellini* (*Pipistrellus* and *Nyctalus*), whereas they were absent from another bat tribe *Plecotinii* (*Barbastella* and *Plecotus*). Within the subfamily *Myotinae*, bats were found to be positive in several species. Except for two *Myotis emarginatus* (MF540608, MF540609), that could represent a group based on clustering according to the analysis of the hexon partial gene, the rest of *Myotis* were sparsely along the phylogenetic trees without making any monophyletic cluster Interestingly, AdVs were not found in some Myotis, *M. daubentonii*, despite this species being well represented in the screening (*n* = 60 and *n* = 41 for OPS and SS, respectively).

Previous studies mostly focused on the analysis of guano and internal tissues [9,10,13,14,17]. The analysis of OPS for the screening of AdV is a novel aspect of this study and has allowed the AdV detection in the upper respiratory tract of bats and highlighted a possible faecal-oral transmission route with the same AdV identified in OPS and SS samples from two *P. pygmaeus* bats (Ppy15_160725 and Ppy14_160725). The phylogenetic reconstructions identify, in both type of samples, AdVs highly related in different groups of bats, supporting this possible oral-faecal transmission. An important reason for the study of OPS in bats is the fact that many human AdV serotypes have not a specific well identified cellular receptor and given that replicate poorly in animals [34], the understanding of factors that define tropism and transmission during a natural infection increase the knowledge of AdV infections. Notably, in bats a possible faecal-oral transmission route is an interesting issue to explore considering bat as emerging and re-emerging infectious diseases vectors.

Previous authors have published new bat mastadenovirus mostly based on the phylogenetic analysis of a short and informative fragment of the DNApol gene [10,11,12,14]. This is a well conserved gene involved in viral transcription [35]. Despite its extensive use in phylogenetic analysis of new human and animal AdVs, the resolution of the phylogenetic reconstruction based on it is limited (less than 100 amino acids). The PCR presented in this study amplified ~450 bp, offering the possibility to increase the resolution of the phylogenetic tree. However, with the aim to compare our sequences with the previously published from the Central Europe [14] and the reference sequences available in the GenBank database, the length was reduced to 277 bp. Currently, ICTV has accepted two bat AdVs species, *Bat mastadenovirus A* [12] and *Bat mastadenovirus B* [13]. According to the taxonomic criteria [8] and based on the distance matrix analysis, the bat mastadenoviruses identified in our study represent potentially new species in the genus *Mastadenovirus* and very divergent from the ICTV references, even with the potentially novel species proposed, with the exception of three detected in *P. pygmaeus* (Ppy15_160725, Ppy21_160725, Ppy14_160725). Moreover, one *P. kuhlii* (Pku2_160622) and one *N. lasiopterus* (Nlas_K01317) were associated with the *Bat mastadenovirus B* although there was greater than 15% difference suggesting new bat AdV species. It is remarkable that bat AdVs obtained from the species *P. kuhlii* and *P. pygmaeus* clustered together in two well supported groups indicating host specificity even at the species level.

In this work, the identification of new bat AdVs is further supported by the results obtained using the hexon gene, a more variable protein [11,12,16,19] which contains seven hypervariable regions identified as viral epitopes [36]. Nucleic acids variation define the different human serotypes [37]. Our generic PCR in the hexon gene was designed in the hypervariable region 7 and the analysis of the sequences obtained were in concordance with the genotype and serotype in human AdVs [30].

The evolutionary relationships based on the two partial genes are presented separately since they provide different information according to their different mutation rates. Both genes agree in the main structure of their tree topologies and clusters and both provide support for a presumably new Iberian bat mastadenoviruses clustering and distinguishing between the families Vespertilionidae and Rhinolophidae in the phylogenies. Most of the available AdVs in the GenBank database grouped within the three monophyletic groups corresponding to their host genera *Pipistrellus*, *Nyctalus* and *Rhinolophus*. This relationship is also supported by the phylogenetic analysis of the DNApol gene in which the AdV detected in a *N. leisleri* (Nle_00954) clusters with a bat AdV detected in a *N. leisleri* sampled in Hungary [14]. In our sampling, more basal relationships among the main bat hosts were more difficult to be affiliated with their hosts due to the lack of representation of important bat groups such as *Scotophillinii*, *Nycticeinii* and *Plecotinii* within the family Vespertilionidae. These host-pathogen relationships were clearly observed with herpesviruses [27] but still, AdVs could represent another example of parallel evolution of DNA virus and their bat hosts. The phylogenetic analysis of partial hexon gene showed no similarities in the sequences between AdVs from bats captured in the South and the North of Spain, as it is shown in a *P. pygmaeus* (Pyi6_070616) collected in Lugo (North) and the *P. pygmaeus* (Ppy15_160725) collected in Seville (South).

Although most of the AdVs clustered by their bat host, some exceptions are clearly remarkable. In the hexon gene, the AdV detected in a *M. myotis* (Mmy8_080623) clustered with the group composed of two different species of *Rhinolophus* bats. It is well known that many *Myotis* colonies share roosts with several species of the genus *Rhinolophus* and this could be the origin of the inter-specific transmission between these two bat species. Secondly, the AdV detected in a *H. savii* (Hsa6_070704), clustered with adenovirus from the Rhinolophus group despite the DNApol gene revealing a specific AdV group in three different *H. savii*. In this second example, a natural transmission seems less likely since the two species have very different life history and barely share any ecological requirement. Nevertheless, the description of recombinant viruses is a common phenomenon in human AdV [38] and could explain the different results. However, this possible recombination in bat AdVs requires a further confirmation by the complete genomic sequence. A third exception showed the AdV detected in a *M. emarginatus* (Mem15-080703) that clustered together with a *Bat mastadenovirus G* detected in a *Corynorhinus rafinesquii* bat and two others detected in Myotis bats from Hungary. The *C. rafinesqii* is a vespertilionid bat, the distribution of which is restricted to the Southeast of North America and Mexico [19,39]. The connection between these viruses is an intriguing given that their hosts are geographically and evolutionary distant, although it could be related to a recent colonization of North America by Palearctic Myotis [40].

In conclusion, based on the analysis of two different regions of genome used to study two different type of samples, the present study contributes with potentially new members from *Mastadenovirus* genus distinct from previously described reference species *Bat mastadenovirus A* and *B* [10,12]. The new AdV groups were detected in bats captured in a broad geographical region and generate data supporting that diversity of bat mastadenovirus is associated by host and the distribution of the host.

## Figures and Tables

**Table 1:** Bat species studied, AdV positive results, year of capture and GenBank accession numbers. ^1^ Abb., Bat species abbreviations, ^2^ OPS, Oropharyngeal swabs, ^3^ SS, Stool samples, ^4^ Capture Year, ^5^ GenBank Accession number for hexon sequences, ^6^ GenBank Accession number for DNA polymerase sequences. N/A: Not available.

Iberian Bat Species		Abb ^1^	OPS ^2^	SS ^3^	Year ^4^	Hexon Sequences ^5^	DNA-Pol Sequences ^6^
Family	Name						
Vespertilionidae	*Barbastella barbastellus*	Bba	0/38	0/4	07,08	N/A	N/A
	*Eptesicus isabellinus*	Eis	0	0/8	04,07	N/A	N/A
	*Eptesicus serotinus*	Ese	0	0/14	03,07	N/A	N/A
	*Hypsugo savii*	Hsa	0/31	3/26	07	HM856338,41,42	JX065121, 22, MG208122
	*Myotis alcathoe*	Mal	0	0/1	07	N/A	N/A
	*Myotis bechsteinii*	Mbe	1/18	0/2	07	MF540611	N/A
	*Myotis blythii*	Mbl	0/29	0	04	N/A	N/A
	*Myotis capaccinii*	Mca	0/15	0	04,07	N/A	N/A
	*Myotis daubentonii*	Mda	0/63	0/41	04,07	N/A	N/A
	*Myotis emarginatus*	Mem	3/56	0	08	MF540608-10	N/A
	*Myotis escalerai*	Mes	0/13	0	04,07	N/A	N/A
	*Myotis myotis*	Mmy	1/79	0/1	04,07	HM856353	N/A
	*Myotis mystacinus*	Mmt	0/2	0/8	07	N/A	N/A
	*Myotis nattereri*	Mna	0/36	0/3	07	N/A	N/A
	*Nyctalus noctula*	Nno	3/122	0	07	MF540597-99	N/A
	*Nyctalus lasiopterus*	Nlas	10/139	6/40	07	HM856327-34,39-40,43,45-47, 50, MG132211	JX065117-20, 23,25-26,28
	*Nyctalus leisleri*	Nle	1/19	3/26	07	HM856344,48, 51-52	JX065124,27,29
	*Pipistrellus kuhlii*	Pku	12/350	2/4	07,16	MF540577-85,87,89	MF404970-73,75,86
	*Pipistrellus pipistrellus*	Ppi	0/29	0/4	07,16	HM856349	N/A
	*Pipistrellus pygmaeus*	Ppy	6/36	11/120	07,16	MF540575-76, 86,88,90-96	MF404968-69,74, 76-79, 80-85,87-89
	*Plecotus auritus*	Pau	0/11	0/8	04,07	N/A	N/A
	*Plecotus austriacus*	Pas	0/10	0/6	04,07	N/A	N/A
Miniopteridae	*Miniopterus schreibersii*	Msc	0/152	0/2	04,07, 16	N/A	N/A
Rhinolophidae	*Rhinolophus euryale*	Reu	6/49	0	04,07, 08	MF540600-02,12-13 HM856335	N/A
	*Rhinolophus ferrumequinum*	Rfe	7/90	1/3	04,07	MF540603-07,14 HM856336-37	N/A
	*Rhinolophus hipposideros*	Rhi	0/4	0/4	07,08	N/A	N/A
	*Rhinolophus mehelyi*	Rme	0/1	0	07,08	N/A	N/A
Total	27/32		50/1392	26/325		69 49OPS + 20SS	35 14OPS + 21SS

**Table 2:** Spanish bat mastadenoviruses classified by the amino acid distance matrix analysis based on partial DNA-dependent DNA polymerase. ^1^ Values more than 15% are potentially new species following the demarcation criteria. Abb: P: *Pipistrellus*. N: *Nyctalus*. H: *Hypsugo*.

Group of Bat AdV	Tentative Virus Name	Abb Name	% aa Pairwise Distances ^1^	
			BtAdV-3	BtAdV-2
bat AdVs associated with BtAdV-2	Bat mastadenovirus *P. pygmaeus* 14 160725	Ppy14 160725	31.4	11.8
	Bat mastadenovirus *P. pygmaeus* 15 160725	Ppy15 160725	31.4	12.7
	Bat mastadenovirus *P. pygmaeus* 21 160725	Ppy21 160725	32	12.2
Potentially novel bat AdVs	Bat mastadenovirus *N. lasiopterus* K01317	Nlas K01317	33.8	25.5
	Bat mastadenovirus *N. lasiopterus* K01508	Nlas K01508	35	41.8
	Bat mastadenovirus *N. lasiopterus* K01530	Nlas K01530	35.7	42.5
	Bat mastadenovirus *N. lasiopterus* K01504	Nlas K01504	35.7	42.5
	Bat mastadenovirus *N. leisleri* 3 070704	Nle3 070704	32.5	42.5
	Bat mastadenovirus *N. leisleri* 00964	Nle 00964	34.5	44
	Bat mastadenovirus *N. leisleri* 5 00954	Nle5 00954	39.7	41.8
	Bat mastadenovirus *N. lasiopterus* K01535	Nlas K01535	41.8	52.1
	Bat mastadenovirus *N. lasiopterus* K00911	Nlas K00911	43.5	46.1
	Bat mastadenovirus *N. lasiopterus* 4 050701	Nlas4 050701	42.6	38.8
	Bat mastadenovirus *N. lasiopterus* 8 050701	Nlas8 050701	42.6	38.8
	Bat mastadenovirus *P. kuhlii* 2 160622	Pku2 160622	37.5	23.8
	Bat mastadenovirus *P. kuhlii* 1 160517	Pku1 160517	39.4	39
	Bat mastadenovirus *P. kuhlii* 15 160622	Pku15 160622	39.4	39
	Bat mastadenovirus *P. pygmaeus* 20 160628	Ppy20 160628	38.7	39
	Bat mastadenovirus *P. kuhlii* 16 160517	Pku16 160517	38.7	38.3
	Bat mastadenovirus *P. kuhlii* 17 160517	Pku17 160517	38.7	38.3
	Bat mastadenovirus *P. kuhlii* 6 160517	Pku6 160517	38.7	38.3
	Bat mastadenovirus *P. pygmaeus* 29 160622	Ppy29 160622	44.8	36.5
	Bat mastadenovirus *P. pygmaeus* 31 160622	Ppy31 160622	41.2	41.6
	Bat mastadenovirus *P. pygmaeus* 23 160628	Ppy23 160628	41.2	41.6
	Bat mastadenovirus *P. pygmaeus* 2 160725	Ppy2 160725	41.2	41.6
	Bat mastadenovirus *P. pygmaeus* 27 160628	Ppy27 160628	41.2	41.6
	Bat mastadenovirus *P. kuhlii* 11 160622	Pku11 160622	42	40.9
	Bat mastadenovirus *P. pygmaeus* 37 160622	Ppy37 160622	42	40.9
	Bat mastadenovirus *P. pygmaeus* 11 160725	Ppy11 160725	42	40.9
	Bat mastadenovirus *P. pygmaeus* 19 160628	Ppy19 160628	42	40.9
	Bat mastadenovirus *H. savii* 6 070704	Hsa6 070704	41	41.7
	Bat mastadenovirus *H. savii* 2 070613	Hsa2 070613	41	41.7
	Bat mastadenovirus *H. savii* 2 070703	Hsa2 070703	41	41.7

## Supplementary Materials

The following are available online at http://www.mdpi.com/1999-4915/10/8/443/s1, Table S1: Tentative virus names, type of sample and localization of *Pipistrellus* group, Table S2: Tentative virus names, type of sample and localization of *Nyctalus* group, Table S3: Tentative virus names, type of sample and localization of Hypsugo and Myotis groups and Table S4: Tentative virus names, type of sample and localization of Rhinolophus group.
