# Supplementary material for: New Adenovirus Groups in Western Palaearctic Bats
Source: Viruses. 2018 Aug 20;10(8):443. doi: 10.3390/v10080443 (PMC6116233; doi:10.3390/v10080443)
Supplement: Supplementary file 1 [file viruses-10-00443-s001.pdf]

Table S1: Tentative viruses names, type of sample and localization of *Pipistrellus* group.

**Tentative virus name**	**Type of sample**		**Localization**
	OPS	SS	
Bat mastadenovirus P.pygmaeus 2 160725		X	Sevilla
Bat mastadenovirus P.pygmaeus 11 160725		X	Sevilla
Bat mastadenovirus P.pygmaeus 14 160725	X	X	Sevilla
Bat mastadenovirus P.pygmaeus 15 160725	X	X	Sevilla
Bat mastadenovirus P.pygmaeus 21 160725		X	Sevilla
Bat mastadenovirus P.pygmaeus 5 160811	X		Sevilla
Bat mastadenovirus P.kuhlii 1 160517	X		Córdoba
Bat mastadenovirus P.kuhlii 3 160517	X		Córdoba
Bat mastadenovirus P.kuhlii 6 160517	X		Córdoba
Bat mastadenovirus P.kuhlii 16 160517	X		Córdoba
Bat mastadenovirus P.kuhlii 17 160517	X		Córdoba
Bat mastadenovirus P.kuhlii 20 160517	X		Córdoba
Bat mastadenovirus P.kuhlii 22 160517	X		Córdoba
Bat mastadenovirus P.kuhlii 40 160517	X		Córdoba
Bat mastadenovirus P.kuhlii 15 160518	X		Córdoba
Bat mastadenovirus P.kuhlii 2 160622	X		Huelva
Bat mastadenovirus P.kuhlii 11 160622		X	Huelva
Bat mastadenovirus P.pygmaeus 15 160622	X	X	Huelva
Bat mastadenovirus P.kuhlii 18 160622	X		Huelva
Bat mastadenovirus P.pygmaeus 19 160622		X	Huelva
Bat mastadenovirus P.pygmaeus 29 160622	X		Huelva
Bat mastadenovirus P.pygmaeus 31 160622		X	Huelva
Bat mastadenovirus P.pygmaeus 37 160622		X	Huelva
Bat mastadenovirus P.pygmaeus 19 160628		X	Huelva
Bat mastadenovirus P.pygmaeus 20 160628	X		Huelva
Bat mastadenovirus P.pygmaeus 23 160628		X	Huelva
Bat mastadenovirus P.pygmaeus 27 160628	X		Huelva
Bat mastadenovirus P.pygmaeus 6 070616		X	Lugo

Table S2: Tentative viruses names, type of sample and localization of *Nyctalus* group

Tentative virus name	Type of sample		Localization
	OPS	SS	
Bat mastadenovirus N. lasiopterus K01076	x		Cádiz
Bat mastadenovirus N. lasiopterus K01093	x		Sevilla
Bat mastadenovirus N. lasiopterus K01081	x		Cádiz
Bat mastadenovirus N. lasiopterus K01089	x		Cádiz
Bat mastadenovirus N. lasiopterus K00911		x	La Rioja
Bat mastadenovirus N. lasiopterus K01024	x		Cádiz
Bat mastadenovirus N. lasiopterus K01502		x	Málaga
Bat mastadenovirus N. lasiopterus K01504		x	Málaga
Bat mastadenovirus N. lasiopterus 4 050701	x		Sevilla
Bat mastadenovirus N. lasiopterus 8 050701	x		Sevilla
Bat mastadenovirus N. lasiopterus K01317	x		Sevilla
Bat mastadenovirus N.leisleri 00954		x	Málaga
Bat mastadenovirus N. lasiopterus K01508		x	Málaga
Bat mastadenovirus N. lasiopterus K01535		x	La Rioja
Bat mastadenovirus N. lasiopterus K01550	x		Málaga
Bat mastadenovirus N.leisleri 00964		x	La Rioja
Bat mastadenovirus N. lasiopterus K01530		x	La Rioja
Bat mastadenovirus N.leisleri 3 070704		x	Gerona
Bat mastadenovirus N.leisleri 1 080616	x		Navarra
Bat mastadenovirus N. noctula 13341	x		Navarra
Bat mastadenovirus N. noctula 13481	x		Navarra
Bat mastadenovirus N. noctula 13483	x		Navarra
Bat mastadenovirus N. lasiopterus K1849	x		Cádiz

Table S3: Tentative viruses names, type of sample and localization of *Hypsugo* and *Myotis* groups

Host	Tentative virus name	Type of sample		Localization
		OPS	SS	
**Hypsugo group**	Bat mastadenovirus H. savii 6 070704		X	Gerona
	Bat mastadenovirus H. savii 2 070613		X	Cáceres
	Bat mastadenovirus H. savii 2 070703		X	Gerona
**Myotis group**	Bat mastadenovirus M. emarginatus 5 080703	X		Vizcaya
	Bat mastadenovirus M. emarginatus 8 080703	X		Vizcaya
	Bat mastadenovirus M. emarginatus 15 080703	X		Vizcaya
	Bat mastadenovirus M. bechsteinii 1 080619	X		Navarra
	Bat mastadenovirus M. myotis 6 080623	X		Córdoba

Table S4: Tentative viruses names, type of sample and localization of *Rhinolophus* group.

Tentative virus name	Type of sample		Localization
	OPS	SS	
Bat mastadenovirus R.ferrumequinum 1 40622	X		Valencia
Bat mastadenovirus R.ferrumequinum 1 070704		X	Gerona
Bat mastadenovirus R.euriale 12 080623	X		Córdoba
Bat mastadenovirus R.euriale 19 080623	X		Córdoba
Bat mastadenovirus R.euriale 5 080623	X		Córdoba
Bat mastadenovirus R.ferrumequinum 19 080623	X		Córdoba
Bat mastadenovirus R.ferrumequinum 23 080623	X		Córdoba
Bat mastadenovirus R.ferrumequinum 25 080623	X		Córdoba
Bat mastadenovirus R.ferrumequinum 5 080623	X		Córdoba
Bat mastadenovirus R.ferrumequinum 23 080707	X		Vizcaya
Bat mastadenovirus R.euriale 25 080623	X		Córdoba
Bat mastadenovirus R.euriale 18 080623	X		Córdoba
Bat mastadenovirus R.ferrumequinum 30 080623	X		Córdoba
Bat mastadenovirus R.euriale 2 40409	X		Valencia
Bat mastadenovirus R.euriale A15	x		Valencia
